# Supplementary material for: Treatment-seeking behaviour among people with opioid use disorder in the high-income countries: A systematic review and meta-analysis
Source: PLoS One. 2021 Oct 15;16(10):e0258620. doi: 10.1371/journal.pone.0258620 (PMC8519451; doi:10.1371/journal.pone.0258620)
Supplement: S1 File — (DOCX) [file pone.0258620.s002.docx]

**S1 File**

**Appendix A**

Search strategy


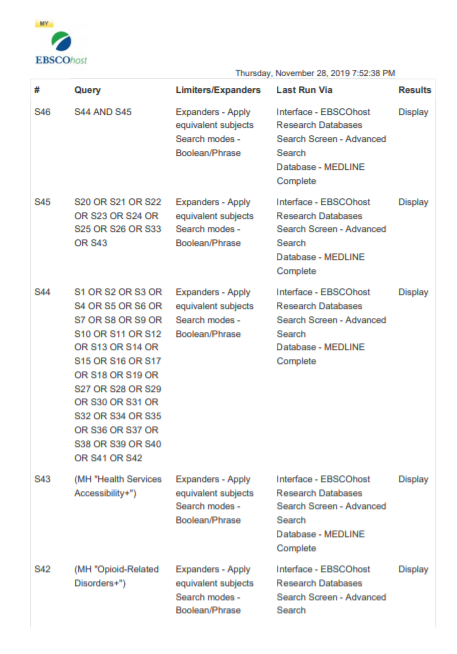


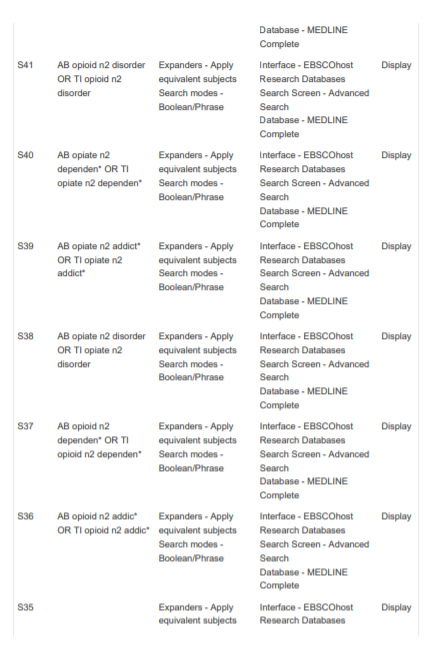


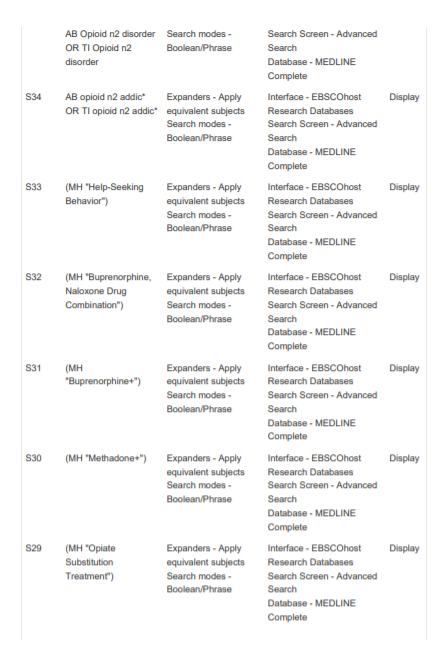


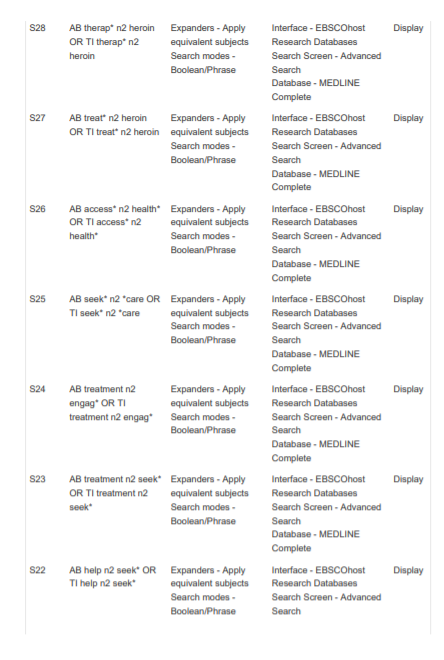


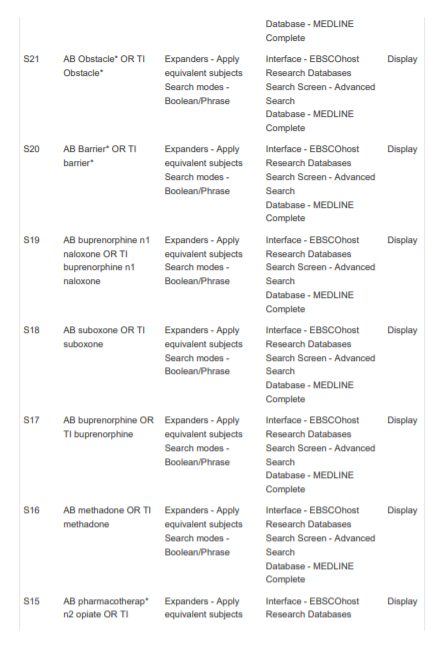


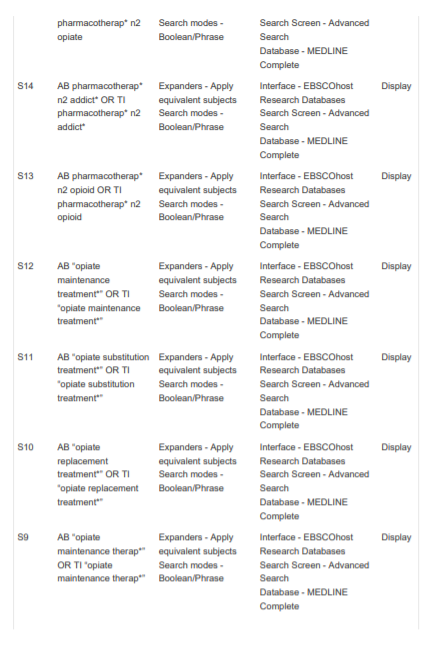


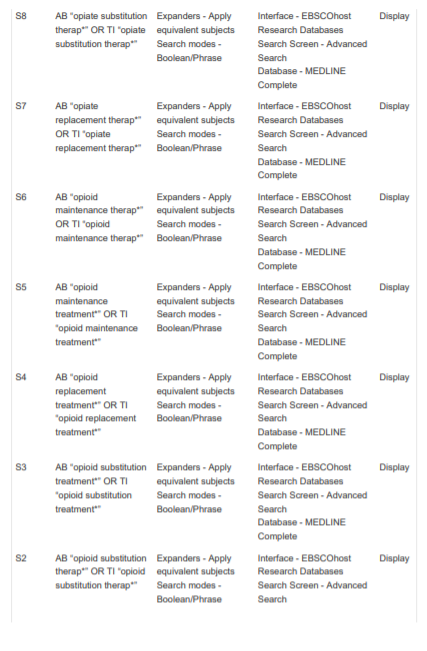


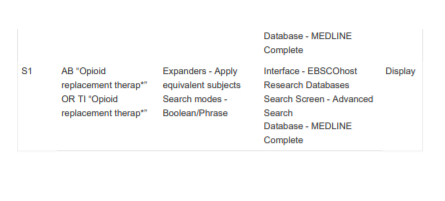


**Appendix B**

Key characteristics of included studies

| **#** | **Author, year, country** | **Study type, sample size** | **Sample description** | **Opioid of use** | **Opioid use disorder screening tool** | **Participant mean age and gender** | **Outcome (treatment accessed)** | **N (%) sought treatment, (SE or CI), timeframe** | **Number of Joanna Briggs Institute quality criteria met** |
| --- | --- | --- | --- | --- | --- | --- | --- | --- | --- |
| **1** | Becker et al, 2008, USA | Cross sectional, n= 1736 | Participants were civilian, non-institutionalized population aged 12 years and older with opioid use disorder. The 2002–2004 National Survey on Drug Use and Health (NSDUH) was the data source | Prescription opioids | DSM-IV criteria for prescription opioid use disorder, heroin use disorder or both | M= not reported  Male = 53.1% | Treatment or counselling for OUD | 15.2% (251/1653), 12-month treatment seeking | 88%  Good |
| **2** | Blanco et al, 2013, USA | Cross sectional, n=623 | Participants were civilian, non-institutionalized population 18 years and older with opioid use disorder. The 2004-2005 National Epidemiologic Survey on Alcohol and Related Conditions II (NESARC-II) was the data source. | Prescription opioids | DSM-IV criteria for prescription opioid use disorder | M= not reported  Male = 56.3%  (N=351) | Treatment from health professional or community agency for OUD | 29.20%, SE=2.15, Lifetime treatment seeking | 88%  Good |
| **3** | Choi et al, 2019, USA | Cross sectional, n=988 | Participants were civilian, noninstitutionalized, US population aged 12+ years who had opioid use disorder.  The 2015–2016 NSDUH provided data. | Heroin and prescription opioids | DSM-IV criteria for prescription opioid use disorder, heroin use disorder or both | M= not reported  Male = 60.8%  (N=601) | Treatment or counselling for OUD | 31.5%, 12-month treatment  53.5%, lifetime treatment seeking | 88%  Good |
| **4** | Feder et al, 2018, USA | Cross sectional, n=3287 | Participants were civilian, noninstitutionalized, US population aged 18+ years who had OUD in the year preceding their interview. The 2010-2014 NSDUH provided data | All opioids | DSM-IV criteria for prescription opioid use disorder, heroin use disorder or both | M= not reported  Male = 60.5% 95% CI (57.0–64.0) | Treatment or counselling for OUD | 29.6% (CI 26.3, 33), 12-month treatment | 88%  Good |
| **5** | Kerridge et al, 2015, USA  * | Cross-sectional, n= 36309  N=330 (for 12 month OUD)  N=688 (for life-time OUD) | Participants were U.S. noninstitutionalized adult  civilian population.  NESARC-III survey provided data | Prescription opioids | DSM-V criteria for non-medical prescription opioid use disorder 12-month and lifetime diagnosis | M= not reported  Male = 47.5%  (n=484) | Treatment from health professional or community agency for OUD | Men – 18.1% (SE=4.08)  Women –17.3% (SE=3.52) for 12-month NMPOUD diagnosis,  12-month treatment  Men-26.8% (SE=2.94)  Women – 31.1% (SE=2.71) for lifetime NMPOUD, lifetime treatment history | 88%  Good |
| **6** | Mowbray et al, 2010, USA | Cross sectional, n=150 (ever used heroin)  N=104 for heroin abuse or dependence | Participants were civilian, non-institutionalized population 18 years and older, with a history (or current) heroin use. The 2001-2002 NESARC was the data source. | Heroin | DSM-IV criteria for heroin abuse and/or heroin dependence | M= not reported  Male = 74.3% (SE= 4.16) | Treatment from health professional or community agency for OUD | DSM-V heroin dependence 81.4% (SE 6.06) and DSM-V heroin abuse 69.2% (SE 7.41), Lifetime treatment seeking | 88% Good |
| **7** | Novak et al, 2019, USA | Cross sectional, n=3398 | Participants were civilian, noninstitutionalized, US population aged 19-64 years with OUD. The 2008-2014 NSDUH provided data | Prescription opioids or heroin | DSM-IV criteria for abuse or dependence | M= not reported  Male = 70% (SE=0.02) | Treatment or counselling for OUD | OUD treatment-Weighted proportion  No mental illness, 0.14 (SE=0.01). Mild mental illness 0.11 (SE=0.02).  Serious mental illness, 0.10 (SE=0.02),  12 months | 88%  Good |
| **8** | Ober et al, 2018, USA | Cross sectional, n=392  Heroin use disorder, n=116  Prescription opioid use disorder, n=61 | Participants (18+) were recruited from a multi-site health centre and were included if opioid and/or alcohol use disorder (OAUD) diagnosis | Heroin and prescription opioids | DSM-IV criteria for abuse or dependence of heroin and/or abuse or dependence of prescription opioids | M= 41.8 (SD=12.0)  Male = 79.1% (n=310) | Initiation of medication assisted therapy for OUD | Heroin use disorder –17.2% initiated MAT (20/116),  PrescriptioOUD –14.8%  initiated MAT (9/61),  6 months | 88%  Good |
| **9** | Saha et al, 2016, USA  ^[[1]](#footnote-1)^ | Cross sectional, n=36309  N=330 (for 12 month OUD)  N=688 (for life-time OUD) | Participants were U.S. noninstitutionalized adult  civilian population. The NESARC-III provided data. | Prescription opioids | DSM-IV and V nonmedical prescription opioid use disorder (NMPOUD) criteria for 12-month and lifetime diagnosis | M= not reported  Male = 47.5%  (n=484) | Treatment from health professional or community agency for OUD | 17.7% (SE=2.8) for 12-month NMPOUD diagnosis,  12-month treatment  28.9% (SE=2.07) for lifetime NMPOUD, lifetime treatment history | 88%  Good |
| **10** | Saloner et al, 2015, USA | Cross sectional N=6770 | Participants were civilian, noninstitutionalized, US population aged 12+ years who had OUD in the year preceding their interview. The 2004-2013 NSDUH provided data | Prescription opioids and heroin | DSM-IV criteria for OUD abuse and/or dependence | M= not reported  Male = 49.4%  (n=3341) | Treatment or counselling for OUD | 16.6% (CI 14.2, 19) for 2004 to 2008  21.5% (CI 19.1 24) for 2009 to 2013, 12-month treatment | 88% Good |
| **11** | Subramaniam et al, 2009, USA | Cross sectional, n=94  Heroin n=53  Prescription opioid, n=41 | Participants (14 - 17 years) with OUD were recruited from a single treatment site in Baltimore. | Prescription opioids and heroin | DSM-IV criteria for abuse or dependence | M= not reported, adolescents  Male = 55.3%  (n=52) | Treatment for opioid use disorder and psychiatric treatment | 75.6% (31/41) for prescription OUD  73.6% (39/53) for HUD, Lifetime treatment history | 63%  Moderate |
| **12** | Wu et al, 2011, USA | Cross sectional, n=789 | Participants were civilian, noninstitutionalized, US population aged 12-17 years who had 12-month NMPOUD. The 2005-2008 NSDUH provided data | Prescription opioids | DSM-IV criteria for past-year prescription opioid abuse or dependence | M= not reported, adolescents  Male = 51% | Any treatment or counselling for OUD | 17.4% (SE 2.26) with opioid dependence  16.1% (SE 2.97) with opioid abuse,  12-month treatment | 88%  Good |
| **13** | Wu et al, 2016, USA | Cross sectional, 6125 | Participants were civilian, noninstitutionalized, US population aged 12+ years who had 12-month OUD. The 2005-2013 NSDUH provided data | Prescription opioids or heroin | DSM-IV criteria for prescription opioid use disorder and/or heroin use disorder | M= not reported  Male= 57.4% (SE=1.13) | Any treatment or counselling for OUD | 19.44% (CI 17.63, 21.4), 12-month treatment | 88% Good |

**Appendix C**

RE base model with OUD lifetime treatment only

**Appendix D**

Doi plot with LFK index for IVhet model for OUD lifetime treatment

**Appendix E**

RE base model with OUD 12-month treatment only

**Appendix F**

Doi plot with LFK index for IVhet model for OUD 12-month treatment

**Appendix G**

Lifetime treatment seeking with heroin plus combined OUD IVhet model

**Appendix H**

Lifetime treatment seeking with prescription OUD IVhet model

**Appendix I**

12-month or less treatment seeking with heroin plus combined OUD IVhet model

**Appendix J**

12-month or less treatment seeking with prescription OUD IVhet model

**Appendix K**

12-month or less treatment seeking with general OUD IVhet model

**Appendix L**

Lifetime OUD treatment seeking IVhet base model outlier removed

**Appendix M**

12-month OUD treatment seeking IVhet base model outliers removed

**Appendix N**

Lifetime OUD treatment seeking IVhet base model adults only

**Appendix O**

Lifetime OUD treatment seeking IVhet base model adolescents only

**Appendix P**

12-month OUD treatment seeking IVhet base model adults only

1. These two studies (Kerridge et al 2015 and Saha et al 2015) used the same sample, therefore only Saha et al 2015 is included in the meta-analysis results [↑](#footnote-ref-1)
