# Supplementary material for: Treatment-seeking behaviour among people with opioid use disorder in the high-income countries: A systematic review and meta-analysis
Source: PLoS One. 2021 Oct 15;16(10):e0258620. doi: 10.1371/journal.pone.0258620 (PMC8519451; doi:10.1371/journal.pone.0258620)
Supplement: Attachment — Submitted filename: Response to PLOS one reviewers 30042021.docx [file pone.0258620.s004.docx]

**Reviewer #1: The revised manuscript entitled “Treatment seeking behaviour among people with opioid use disorder in high income countries: A systematic review and meta-analysis” is significantly improved from the original submission. The authors have been comprehensive in their response to prior reviews. I have no additional major critiques. One suggestion would be to state in the abstract that while the search was intended to capture all high income countries only manuscripts from the US met inclusion criteria.**

Thank you for this comment, we have included a line in the results section of the abstract to indicate that only US countries met the inclusion criteria.

Page 2, line 36: “There were 13 quantitative studies included in the systematic review, with all studies being from the United States of America (USA).”

**Reviewer #2: Upon re-reviewing this article I realized that the authors seem to use "treatment seeking" and "access to treatment" interchangeably. For instance, the Objective in the Abstract is to "To determine treatment seeking behaviour in those with opioid use disorder (OUD) in the high-income countries" but the authors present access to treatment. I do not think these 2 terms can be used interchangeably. It would be important for the authors to define/how they conceptualize "treatment seeking" and justify why they then move on to using the term "access". Alternatively, please remove the "access to treatment language".**

We have removed all sections that discuss access to treatment and instead used treatment seeking.

These changes are found:

Abstract page 2, line 44: “Conclusion: This review found that one in five people with OUD sought OUD treatment in the previous 12 months and two in five people with OUD sought OUD treatment in their lifetime.”

Page 4, line 62: “An Australian treatment history study of heroin dependence, found that 88% had previously sought treatment in their lifetime. (21)”

Page 6, line 109: “The data extraction sheet included the following categories to describe and compare the studies: author, year, country, sample description, study design, sample size, opioid type, OUD diagnosis tool, participant mean age and gender, type of treatment sought, treatment time frame and percentage or proportion that sought treatment.”

Page 17, line 309: “It is expected that treatment seeking occurs at a higher proportion within a longer timeframe (12 months versus lifetime) and other studies have also found that increased disease duration and severity increase the likelihood of treatment seeking for OUD.”

Page 19, line 357: “Treatment programs, differing health systems, stigma in differing culture as well as stigma surrounding specific drug used all play a role in treatment seeking behaviour.”

Page 22, line 435: “which may lead to differences in treatment seeking behaviours for those with OUD.”

**Please also specify whether any relevant articles published before 2008 appeared in your search. If so, why were they excluded given that there were no year restrictions for your search.**

We had no restrictions on publication year, however there were no relevant studies included in our review that were published prior to 2008. See Appendix B in the supplemental file for included studies.

**Reviewer #3: The authors were very thoughtful in their responses to the reviewers. However, I still have significant concerns about the methodology that were not alleviated with inclusion of several items as limitations, but rather, call into question the appropriateness of the methodology. Some further detail and considerations may be warranted:

The authors note in their response that “only studies from the USA were available in the literature.” I believe there are still a number of issues that arise from this methodology and the objectiveness of this statement. Despite noting it in the limitations, the fact that only English language publications were used discounts a substantial amount of literature in high-income countries, and so continuing to use the title “High-income countries” is misleading to readers. In addition, there are nuances to other high income countries that may not be accounted for. For instance, were detoxification programs included? These are often referred to as simply ‘substance use detoxification’ programs. Would this be accounted for in the search criteria? Were studies on the prescribing of buprenorphine included? The Canadian Institute for Health publishes treatment-seeking statistics (i.e., proportion of Canadians starting opioid therapy) through assessments of prescriptions due to their ability to follow individuals through national health systems. It’s possible that the studies in the United States dominated the results because without nationalized healthcare, we rely on studies, as opposed to utilization of nationalized health systems for this information, which may see publication elsewhere than a study-based journal article. These are not just limitations, but raise questions about the appropriateness of this methodology. This could be why 11 of the 13 articles includes are from just two national studies in the US, which also raise questions about the generalizability of the information provided.**

Search terms regarding treatment seeking were broad such as treatment seeking, help seeking and heroin n2 therapy, which would include and capture all treatments for opioid use disorder, including detoxification episodes if it were for opioids. We did not include substance use detoxification as one of our search terms as we are focusing specifically on opioid use disorders and not on general substance use disorders. It worth noting that we did include specific search terms such as methadone, buprenorphine and suboxone and therefore buprenorphine prescribing was included. Please note the full search strategy can be found in Appendix A in the supplemental file.

Furthermore, there are no systematic reviews or meta-analysis published in the literature that focus on the treatment seeking proportion for substance use disorders more generally. The WHO publish a review focusing on unmet treatment need for mental health disorders and alcohol dependence but this review did not include any substance use disorders (general or specific). (Kohn, Saxena, Levav, & Saraceno, 2004)

A line in the discussion has been included on page 21, line 425 has been included referring to this:

“Furthermore, there are no reviews looking at treatment seeking in substance use disorders more generally indicating this is an area for further research.”

We have already included a comparison between alcohol dependence from the above stated WHO review (Kohn et al., 2004) and our OUD treatment seeking rates in the discussion. This is found on page 17, line 305 (not highlighted as it was already in the paper):

“The treatment gap for OUD is therefore comparable to the treatment gap for alcohol dependence and higher than the treatment gap for major depression in the Americas. The 12-month treatment gap for alcohol dependence and major depression in the Americas was 73% and 57% respectively.”

Regarding other comments and the inclusion of national statistics such as OST national prescribing in Canada, we have followed systematic review/meta-analysis protocol and included grey literature searching in google using the term ““treatment-seeking behaviour in opioid use disorder” and the first 5 pages (50 articles in total)  **As noted in the previous review, NSDUH and NESARC are both interviews where an individual is asked to self-report behaviors, although the authors have updated the manuscript to note that they are asked specific criteria, here off the DSM. This leads to question of what the authors deemed to be “self-report” vs. “diagnosed” OUD that may have led to studies from other countries being excluded. Many other countries likely do not use the DSM, so there may be variations in how opioid use disorder is clinically diagnosed. In France, OUD is primarily treated by general practitioners who prescribe buprenorphine, and like Canada, this is tracked through the French National Health Insurance database. Did the authors only take articles where a diagnostic process was specifically outlined, or was a physician prescribing buprenorphine for OUD enough, on the assumption that the physician would not prescribe buprenorphine if there was not a clinical diagnosis? More information on needs to be provided on what constituted a “clinical diagnosis.”**

Thank you for this comment. We have gone through the studies that were excluded from the meta-analysis due to “no appropriate/consistent measure of OUD ie) self-report, injecting marks.”

There were 8 studies that were excluded because there was “no appropriate/consistent measure of OUD ie) self-report, injecting marks” and these were the following studies (the way in which OUD was “diagnosed” is also given):

1) Larance 2019 – Participants in this study were included if they used a S8 opioid that was given to them legally from a pharmacy in the last 6 weeks.
2) Liebling 2016 – Participants in this study were included if they reported non-medical use of prescriptions opioids in the past 30 days
3) Lundgren 2005 – Participants were included in this study if they reported injecting drugs at least once (primarily heroin) in the year prior
4) Oppenheimer 1988 – Participants were obtained from a drug dependence clinic in South East London. No reference to other inclusion criteria regarding review by doctor, whether they were current or past attendees of the clinic (some participants were interviewed whilst in prison so they could not be current clinic users)
5) Robles 2006 – Participants were included in the study if they reported injecting drugs or smoking crack cocaine during the past 30 days.
6) Stein 2018 – 1 Medicare claim for methadone or buprenorphine, this is inclusion based on treatment rather than a diagnosis and therefore all had experienced treatment and therefore there is no relevant treatment seeking proportion. In this case this study should be changed to the group “no appropriate treatment seeking proportion given.”
7) Stumbo 2017 - minimum of two opioid dependence diagnoses recorded in the EMR during an encounter in the 12 months preceding recruitment. It is common to require two instances of a diagnosis when identifying a sample from the EMR as it greatly reduces misidentification based on a single instance of the diagnosis. As there are electronic medical records present for these participants, this shows that the participants are already likely to be engaged in treatment and therefore this study should also be moved into the “no appropriate treatment seeking proportion given.”
8) Yang 2011 – Participants were included in this study if they reported illicit drug use in the past month.

Studies 1 to 3, study 5 and study 8 all report opioid use in a period of time and there is no assessment by a doctor to indicate the participants have OUD. These studies should stay in the exclusion criteria “no appropriate/consistent measure of OUD i.e.) self-report, injecting marks.”

Study 4 includes participants if they were seen at a dependence clinic, however there is no reference to participants having seen or diagnosed with OUD by a doctor. Furthermore, the participants could be current or past users of the clinic indicating previous but not current use of opioids. This study should stay in the exclusion criteria “no appropriate/consistent measure of OUD i.e.) self-report, injecting marks.”

Study 6 and 7 involve Medicare treatment claims or opioid diagnoses on electronic medical records. This indicates that all participants have already had engagement with the healthcare system and therefore these studies should be removed from the category “no appropriate/consistent measure of OUD i.e.) self-report, injecting marks” and included in “no appropriate help-seeking numerical value related to treatment seeking.” Therefore, we have changed the PRISMA and removed 2 papers from the “no appropriate/consistent measure of OUD i.e.) self-report, injecting marks” and this category now has 7 papers in it and the “no appropriate help-seeking numerical value related to treatment seeking” category now has 150 papers. Prisma has been updated, see figure 1.

We have included an additional author in the paper, who helped with revision comments and review. She is Professor Maree Teesson, director of the Matilda Centre for Research in Mental Health and Substance Use, Sydney University. I have included her in the unblinded title page.

**References**

Kohn, R., Saxena, S., Levav, I., & Saraceno, B. (2004). The treatment gap in mental health care. *Bulletin of the World health Organization, 82*(11), 858-866. Retrieved from <https://www.scielosp.org/article/bwho/2004.v82n11/858-866/en/>
