# Supplementary material for: Treatment-seeking behaviour among people with opioid use disorder in the high-income countries: A systematic review and meta-analysis
Source: PLoS One. 2021 Oct 15;16(10):e0258620. doi: 10.1371/journal.pone.0258620 (PMC8519451; doi:10.1371/journal.pone.0258620)
Supplement: Attachment — Submitted filename: Rebuttal letter and all manuscript changes_NH+LL+CM.docx [file pone.0258620.s005.docx]

Thank you for re-considering our manuscript in PLOS ONE, we appreciate the time and effort spent on reviewing our paper. While we normally respect editorial decisions regarding paper rejection, we feel the paper adheres to the principles of publication for PlosOne, which are largely centred around methodological rigour, so we are pleased that our appeal request has been approved. The paper has been reviewed by three separate reviewers and has now been revised three times by us. Upon the first revision we were told that reviewer #1 and reviewer #2 generally agree with the publication of the paper, but that reviewer #3 raised issues surrounding search criteria terms, national prescribing statistics in Canada and how OUD was deemed “diagnosed” versus “self-reported”, that needed to be further addressed. The second revision therefore focused on reviewer #3 comments, which we addressed, and believe are now resolved. The final revision focussed on using treatment seeking consistently throughout the paper, rather than interchanging this with treatment gap (which are not the same) and paying special attention to concerns regardingthe inclusion of US-based studies only. We strongly believe that we have written a methodologically and technically sound systematic review and meta-analysis, which follows the PRISMA guidelines that is the requirement for publication in PLOS ONE and have addressed all reviewer comments and resolved reviewer issues. Please see below for a detailed point-by-point response to all the concerns raised by the reviewers at each revision and the changes made to the manuscript at each revision.

***1^ST^ REVISION***

**Reviewer #1: The current manuscript entitled “Treatment seeking behaviour among people with opioid use disorder in high income countries: A systematic review and meta-analysis” evaluates treatment seeking behavior in individuals with OUD. The protocol for the systematic review and meta-analysis appear sound and maintain rigor (although see point 1 below). The findings are interesting and add to the body of evidence that healthcare providers and programs need to do more to reduce barriers to OUD care and medication for OUD. There are a number of weakness that diminish enthusiasm and need to be addressed should the authors be invited to submit a revision. Most importantly, there is one article that was included but does not appear to meet inclusion criteria (see point 3 below). This may change the results and limits the scope to US populations. There are also some stigmatizing elements of the discussion that are unsupported or based on extremely outdated evidence. Major and minor issues detailed below.

Major
1) The manuscript stated that the search was performed on 11/28/2019 but the PROSPERO protocol was created on 04/20/20. This alone is not necessarily a problem, but registering after starting literature search is not consistent with PRISMA guidelines. The authors should state that the protocol was pre-registered after search was completed and clearly state what aspects of the protocol were completed prior to registration on PROSPERO. It is possible that registration was in response to a prior review at another journal, but the authors should be transparent.**

Response: Please note that the PROSPERO protocol was submitted to PROSPERO on 25^th^ November 2019 (this date is confirmed by an email from PROSPERO). The 20^th^ April 2020 is the date the protocol was accepted for registration on PROSPERO. The delay in registration is potentially due to COVID-19.

**2) Please consider using “medications for Opioid Use Disorder” instead of OST or, to better describe buprenorphine and methadone, use opioid agonist treatment.**

Thank you, this is a good point and OST has been changed to opioid agonist treatment or OAT in numerous places of the manuscript. See page 4, line 58 and page 6, line 102 line 103 and line 104.

**3) The authors state that only studies that included DSM-5 or ICD diagnosed opioid use disorder were included and studies that relied on self-reported OUD were excluded. However at least one study (Kimergard et al., 2017) appears to have used online surveys from social media and treatment websites. It is easily assumed that the participants in these surveys self-reported OUD because the surveys were online links. In fact, the authors state that Kimergard used the Severity of Dependence Scale which is 5 items and, while potentially correlated with DSM and ICD OUD diagnosis, does not equate to an OUD diagnosis. Without this study, all the other studies are in the US which would change the scope of the article (i.e., not high income countries but rather just U.S.). This manuscript would appear to call into the full-text exclusion category of “OUD not appropriately/consistently measured”.”**

This is a valid point and therefore we have removed the Kimergard et al 2017 study from the meta-analysis. The Kimegard et al, 2017 study was removed in the full text exclusion because OUD was not appropriately/consistently measured using the DSM or ICD and instead the severity of dependence scale was used to measure opioid dependence, which does not equate to an OUD diagnosis. The removal of Kimegard et al, 2017 has led to a number of changes in the review which will be discussed below:

Title of the manuscript:

Although the study only includes studies from the USA, the systematic review and meta-analysis did look for treatment seeking in all high-income English speaking countries and therefore the title will not change. A finding of the systematic review and meta-analysis is that all the eligible studies are from the US. We have stated in the abstract in the results and the conclusion that only studies from the USA ended up being included in the review:

Results section of abstract, page 2, line 36:

“Results: There were 13 quantitative studies included in the systematic review, with all studies being from the United States of America (USA).”

Conclusion section of the abstract, page 2, line 44:

“Conclusion: All included studies in this meta-analysis were from the USA. Therefore, this review found that in the USA one in five people with OUD sought OUD treatment in the previous 12 months and two in five people with OUD sought OUD treatment in their lifetime. These results indicate modest levels of treatment seeking for those with OUD in the USA, the reason for this low treatment seeking requires immediate attention and future work.”

Page 2, line 36 abstract results section changed to reflect the removal of the Kimegard et al 2017 study.

“There were 13 quantitative studies included in the systematic review, with all studies being from the United States of America (USA). IVhet models showed that 40% (95% CI: 23%, 58%) and 21% (95% CI: 16%, 26%) sought treatment in their lifetime and past 12 months respectively.”

Figure 1: Prisma flow diagram has been updated to reflect the removal of kimegard et al, 2017. 13 studies are now included in the meta-analysis. Kimegard et al, 2017 study was removed in the full text exclusion because OUD was not appropriately/consistently measured.

Page 8, line 168:

“All 12 studies were conducted in the USA. Most studies (10/12) used nationally representative cross-sectional surveys in the US including the National Survey on Drug Use and Health (NSDUH) and the National Epidemiologic Survey on Alcohol and Related Conditions (NESARC).”

Page 8, line 175:

“Participant recruitment of the remaining two studies occurred via multi-site and single site health care facilities. Eleven of the studies reviewed OUD in adults and two of the studies included only adolescents in the sample.”

Page 9, line 171: Appendix B updated to remove Kimegard et al, 2017 from the key characteristics of included studies table.

Page 9, line 177:

“Most of the included studies (11/12), were of good quality according to the JBI checklist. The remaining study was of moderate quality.”

Multiple changes have been made to account for the removal of Kimegard et al 2017 study:

Page 9, line 186:

“The RE model (Appendix C) and IVhet models produced similar results and therefore the IVhet model will be used. Figure 2 shows that 40% (95% CI: 23%, 58%) of people with OUD sought help for OUD treatment in their lifetime. The I^2^ statistic for heterogeneity is 98%, which suggests a considerable amount of difference between the studies. The five studies included in the lifetime treatment seeking base group were cross-sectional studies from online recruitment samples (33) or nationally representative surveys. (15,18, 20, 34) Two of the studies determined help-seeking behaviour for prescription OUD, (15, 34) the remaining two studies reviewed OUD help-seeking behaviour for all opioids (18) and heroin only. (20) One of the included studies was undertaken in adolescents only (33) and in the remainder of the studies the population was adults. The LFK index, which reports publication bias, reported no asymmetry with LFK = -0.14. (see Appendix D).

Page 9, line 186 reference to Appendix C:

*A new RE lifetime OUD treatment base model with the Kimegard et al, 2017 study removed has been put into Appendix C in the supplemental file

Page 10, line 197 reference to Appendix D:

*A new doi plot with LFK index for IVhet model for OUD lifetime treatment, with the Kimegard et al, 2017 study removed, is included in Appendix D in the supplemental file

Page 9, line 187 reference to Figure 2:

*A new Figure 2 will be uploaded to represent the meta-analysis without the Kimegard et al., 2017 study

Page 11, line 220:

“The treatment-seeking proportion was 54% (95% CI: 26%, 82%), with a high I^2^ statistic (90%).”

*Appendix G (reference to this on page 11, line 220) will be updated to reflect the removal of Kimegard et al 2017.

Page 12, line 224 reference to Appendix H:

*The IVhet model in Appendix H for lifetime treatment seeking with prescription OUD has been updated so that Kimegard et al, 2017 study is removed.

Page 12, line 246 – results have been updated without the Kimegard 2017 study.

“The help-seeking percentage, 39%, (95% CI: 23%, 56%) did not change with removal of the outlier, due to the small weight (2%) the outlier had on the overall pooled proportion. I2 statistic was 98%, indicating significant heterogeneity. The outlier study included individuals with heroin OUD, used a nationally representative sample and had a small sample size (n=150). The included studies were related to prescription OUD, and general OUD. Three of the included studies were nationally representative samples and the remaining study was a single site sample.”

Page 12, line 236, reference to appendix K:

*The IVhet model in Appendix K for lifetime OUD treatment seeking base model with outlier removed has been updated so that Kimegard et al, 2017 study is removed.

Page 13, line 265 – results have been updated without the Kimegard 2017 study

“Four studies were included when the sensitivity analysis was adults only lifetime treatment seeking (see Appendix N). The pooled percentage of treatment seeking was 40% (95% CI: 22%, 59%), with significant heterogeneity (I^2^ =98%). The included studies were identifying treatment seeking in prescription OUD, heroin OUD and general OUD. Four of the five included studies used nationally representative samples and the remaining study used an online opportunistic sample.”

Page 13, line 266 reference to appendix N:

The IVhet model in appendix N for lifetime treatment seeking base model adults only has been updated so that Kimegard et al, 2017 study is removed

Page 15, line 296 shows the updated metaXL sensitivity analysis with Kimegard et al 2017 study removed. The pooled proportion lifetime treatment seeking was 31% to 45%.

“The pooled proportion lifetime treatment seeking was 31% to 45%.”

***Table 1. Lifetime OUD treatment seeking IVhet base model metaXL sensitivity***

| Removed each study in turn | Pooled prevalence | LCI 95% | HCI 95% | Cochran Q | P | I^2^ | I^2^ LCI 95% | I^2^ HCI 95% |
| --- | --- | --- | --- | --- | --- | --- | --- | --- |
| Blanco 2013 | 0.438 | 0.201 | 0.683 | 140.864 | 0.000 | 97.160 | 95.348 | 98.267 |
| Choi 2019 | 0.312 | 0.163 | 0.472 | 62.104 | 0.000 | 93.559 | 87.877 | 96.578 |
| Mowbray 2010 | 0.391 | 0.231 | 0.557 | 145.355 | 0.000 | 97.248 | 95.513 | 98.312 |
| Saha 2016 | 0.445 | 0.208 | 0.689 | 131.828 | 0.000 | 96.966 | 94.977 | 98.167 |
| Subramaniam 20091 | 0.399 | 0.218 | 0.587 | 183.388 | 0.000 | 97.819 | 96.565 | 98.615 |
| Subramaniam 20092 | 0.402 | 0.225 | 0.587 | 179.294 | 0.000 | 97.769 | 96.475 | 98.588 |

In the discussion section, the following changes have been made to reflect the removal of the Kimegard et al, 2017 study (this was the only study from the UK):

Page 17, line 304

“This systematic review and meta-analysis found that treatment-seeking behaviour for OUD was low in the USA”

Page 18, line 348: the removal of the Kimegard 2017 study, which was the only study from the UK, means that all studies are now from the USA. Hence the paragraph now states:

“All studies in this systematic review were from the USA. It is noteworthy that other high-income countries report proportion of seeking treatment in substance use disorders in general, but not specific to opioid use disorders.”

page 19, line 354:

“Therefore this review, with studies only from the USA, found that less people with an OUD sought treatment (one in five people with OUD sought treatment in the past 12 months) than those with SUD in Australia (one in three people with SUD sought treatment in the past 12 months).”

Page 20, line 377:

“The heterogeneity in most of the models was high and contributing factors are variability in OUD type, age of participants and methodological differences.”

Page 21, line 414:

“The low levels of treatment seeking found in the USA in this systematic review are likely a combination of inadequate access to treatment programs and psychosocial challenges, which differ between countries with different health systems.”

Page 22, line 439:

“All included studies were from the USA, which means the results cannot be generalised to other countries due to the notable differences in health care systems between which may lead to differences in access to treatment-seeking behaviours for those with OUD. In the USA healthcare is a market driven business opportunity whereas in other countries for example the UK healthcare is a public good. It would be ideal to include other countries with varying health systems in the review, however presently these studies are not available in the literature.”

Page 23, line 455:

“This review only included studies that were conducted in developed countries and all studies were from the USA; therefore, results are not transferable to countries other than the USA. More research is required on the treatment seeking levels in other countries.”

Page 24, line 475:

“These results indicate low levels of treatment seeking for those with OUD in the USA.”

**4) “Heroin use disorder” is not a diagnosis; OUD with primary heroin use is more appropriate.** **The authors also separate findings into prescription OUD, heroin OUD, and general OUD. This may be unnecessarily confusing. It appears the difference between prescription OUD and non-prescription OUD (i.e. heroin and/or general OUD) is most informative with respect to treatment engagement. Also, the heterogeneity in the prescription OUD is substantially less than the other OUD categories. This is potentially useful and should be discussed more that it is in the findings. Why are the rates of treatment higher and more uniform in prescription OUD versus other OUD categories?**

Heroin use disorder has been deleted and replace with OUD with primary heroin use in two sections, these are page 11, line 219 and page 20, line 380

We have now reclassified OUD into 2 groups that may be less confusing and therefore beneficial as suggested by the reviewer. Group 1 will be prescription OUD and group 2 will be general OUD including OUD with primary heroin use plus combined OUD (where general OUD includes all opioid use). Secondly, an American study reviewing treatment outcomes with prescription OUD, heroin OUD and mixed OUD found that prescription only OUD had more negative urine samples for opioids than heroin OUD and combined OUD. This study also found that prescription OUD participants had longer periods of abstinence from opioids than combined OUD participants.

Please note that lifetime and past 12 months prescription OUD had the lowest treatment seeking rates, but they also had less heterogeneity and narrow confidence intervals indicating more uniform rates of treatment. I have included a sentence discussing the heterogeneity and therefore more uniform treatment in prescription OUD.

Page 20, line 378

“Heterogeneity was low (I^2^= 0%) in two models, which evaluated prescription OUD during the past 12-months and lifetime. Lifetime treatment-seeking in those with OUD with primary heroin use plus combined OUD was the highest, but also had high heterogeneity (I^2^=90%), a wide confidence interval and included only two studies in the meta-analysis, with one study reporting both prescription OUD and heroin OUD. These results imply that treatment seeking is more uniform in those with prescription OUD compared to heroin OUD. In the USA prescription OUD often predates heroin OUD, (59, 60) which may allow health practitioners to notice and target prescription OUD in a more uniform way compared to if the client progresses to heroin OUD. Furthermore, one American study found that the prescription OUD group reported higher rates of other prescription drug abuse than the heroin OUD group. (12) The more regular and continuous contact with health professionals to access prescription medications could further explain the more uniform treatment seeking patterns in those with prescription OUD.”

Please note there is a section on page 7, line 125 explaining this with references:

“Sub-group analysis involved separating prescription OUD and general OUD (heroin OUD and combined OUD). These distinct groups were decided because treatment outcomes with prescription OUD were found to differ from heroin OUD and combined OUD in terms of more negative urine samples and longer periods of opioid abstinence for prescription OUD compared with heroin and combined OUD. (26) Furthermore, the treatment seeking behaviour and perceived need for treatment was higher for those with heroin OUD (including combined OUD) than for those with prescription OUD. (17, 18)”

Changing the subgroup analysis into two groups: prescription and heroin plus combined OUD has led to several changes in the manuscript. These can be found below:

Page 11, line 216

“These subgroups included prescription OUD and heroin plus combined OUD. These two subgroup analyses were done within the two base groups.

***Lifetime treatment seeking***

Lifetime treatment-seeking proportion of those with OUD with primary heroin use plus combined OUD included 3 studies (18, 20, 34) (Appendix G). The treatment-seeking proportion was 54% (95% CI: 26%, 82%), with a high I^2^ statistic (90%). Two of the studies used national surveys and included adults (18, 20) and the third was a single treatment site including only adolescents. (34)”

*Appendix G meta-analysis model in the supplemental material has been updated to include the combined opioids study, Choi et al 2019.

Page 12, line 227

“Six studies (16-19, 32, 36) examined 12-month treatment seeking with heroin plus combined OUD. The treatment seeking for those with heroin plus combined OUD over the past 12 months was 22% (95% CI: 17%, 28%, I^2^ =98%). (Appendix I) One study examined heroin OUD only (32) and five studies examined combined OUD. (16-19, 36). Five studies used the NSDUH survey and the remaining study used a multi-site sample. (32) All six studies included adults only.”

*Appendix I has been updated and is now 12-month or less treatment seeking with heroin plus combined OUD IVhet model

**5) The study column in Table 1 and 2 states “Excluded Study.” These studies were not excluded though. Please clarify.**

This is the metaXL sensitivity analysis, which gives the results of the pooled prevalence if the specified study is removed. I have changed the terminology from excluded study to “removed each study in turn” in tables 1 and 2 and I have also described this sensitivity analysis on page 15, line 294:

“Table 1 show the metaXL sensitivity analysis where the pooled proportion is given when the specified study is removed in turn.”

and page 16, line 299:

“Table 2 show the metaXL sensitivity analysis where the pooled proportion is given when the specified study is removed in turn.”

**6) Discussions about treatment gap for the Europe are inappropriate because there is only one study (only UK and Ireland) and it appears this study should be excluded (see point 2 above). Even if the authors can justify including Kimergard, this single study is in no way representative of Europe.**

Yes, the Kimegard study has been removed and all references to it and to Europe have been removed (see above). In relation to the discussion specifically, the following has been changed:
page 19, line 374

“More research on OUD specific treatment seeking in countries other than the USA is urgently required to better understand treatment seeking within differing countries with differing health systems.”

In the discussion, reference to differing health care systems between the USA and UK has been removed as the Kimegard study is no longer included and all studies are from the USA only

Page 21, line 414

Reference to “high income countries (USA and UK)” **has been removed** and replaced with “the USA”

Page 22, line 439

“All included studies were from the USA, which means the results cannot be generalised to other countries due to the notable differences in health care systems, which may lead to differences in access to treatment-seeking behaviours for those with OUD.”

**7) The authors postulate that individuals with higher rates of OUD and primary heroin use are higher than prescription OUD because of the “heavy disruption of heroin use” on daily life. This is not always the case and potentially not related at all to treatment engagement. The authors also state that “heroin users often seek treatment after hitting ‘rock bottom’ and treatment is often initiated after court orders.” This language is incredibly stigmatizing and potentially inaccurate. In fact, the authors cite a paper from 1971 to justify this statement. Higher rates of treatment seeking for individuals with OUD and primary heroin use may be more likely due to stigma of heroin use and challenges diagnosing prescription OUD particularly in the context of long term opioid treatment for chronic pain (see Manhapra et al. (2020). Complex Persistent Opioid Dependence with Long-term Opioids: A Gray Area That Needs Definition, Better Understanding, Treatment Guidance, and Policy Changes. Journal of General Internal Medicine, 1-8.).**

Thank you for these points and for bringing our attention to this. We have removed the stigmatizing section and replaced it with:

Page 17, line 320

“Reduced treatment seeking in those with prescription OUD when compared with heroin OUD could be explained by the difficulty in diagnosing prescription OUD due to co-existing complex pain management needs and long term opioid use.” (39)

**8) The authors state that individuals with prescription OUD are more likely to have comorbidities compared to those with OUD and primary heroin use. The type of comorbidities should be discussed. Medical comorbidities in individuals with prescription OUD may be driven by co-occurrence of chronic pain, which is associated with considerable multimorbidity.**

We have updated this section to state the specific co-morbidities, which were higher anxiety scores and higher rates of antidepressant prescribing, in those with prescription OUD compared to heroin OUD. We have also included a sentence discussing how depression as a comorbidity can exacerbate the experience of pain.

Page 18, line 332:
“Those with prescription OUD were also more likely to access health services, be prescribed antidepressants, use benzodiazepines, were older and had higher anxiety scores than those with heroin OUD.”

Page 18, line 337

“Furthermore, depression as a comorbidity can exacerbate the experience of pain, leading to increased need for pain relief.”

**9) The authors discuss lower treatment engagement in the current findings compared to general SUD in other developed nations. The authors fail to discuss possible reasons beyond different access to care across countries. This appears to be a great opportunity to talk about potential sigma as a barrier to treatment that may be specific to OUD and review ways to reduce barrier.**

We have included a section discussing how stigma was found to be the third most common reported barrier to OAT

Page 18, line 339:

“A recent systematic review found that stigma was the third most common reported barrier to OAT. (47)”

We have included a section relating to stigma within different cultures and the way OUD is treated in different context:

Page 19, line 365

“In contrast to the USA, European countries, such as Germany and Portugal, have decriminalised drugs for personal use, have medically supervised injecting rooms and have many OUD treatments (including heroin assisted treatment) covered by public health insurance. (53-55) These steps have been shown to increase treatment uptake for OUD. (53, 54) The treatment of OUD as a health condition by these countries has the potential to further reduce stigma by improving community understanding of addiction. (49)”

We have included a section relating to stigma within different classes of drugs ie) alcohol versus opioids

Page 19, line 370

“Furthermore, stigma is experienced differently with different drugs. Internalised stigma is heightened in heroin users compared with marijuana, (56) alcohol and methamphetamine users. (57) Perceived stigma is heightened towards those who use opioids non-medically compared to alcohol, marijuana, ecstasy, and other stimulant users. (57, 58)” **Minor
10) There are numerous awkward phrasing and grammatical issues in the text, most notably in the results. For example, in the results “Lifetime treatment-seeking proportion of those with heroin use disorder included 2 studies only and the treatment seeking proportion was 65% with a wide confidence interval as well as high I2 statistic, both indicating heterogeneity between studies.” This sentence contains a lot of information and is very difficult to follow. Suggest the authors structure reporting the findings in the same way and stick to a consistent presentation of findings. It is sometimes confusing which studies are connected to a reported I2 statistic.**

Thank you, we have changed several sentences throughout the paper to make them grammatically correct and less awkward. They are as follows:

Page 4, line 63

“An Australian treatment history study of heroin dependence, found that 88% had previously accessed treatment in their lifetime.”

Page 9, line 186

“The RE model (Appendix C) and IVhet models produced similar results and therefore the IVhet model will be used.”

Page 9, line 188

“The I^2^ statistic for heterogeneity is 98%, which suggests a considerable amount of difference between the studies.”

Page 11, line 202

“Figure 3 shows that of those with OUD, 21% (95% CI: 16%, 26%) sought treatment in the past 12 months.”

Page 11, line 219

“Lifetime treatment-seeking proportion of those with OUD with primary heroin use plus combined OUD included 3 studies (Appendix G). The treatment-seeking proportion was 54% (95% CI: 26%, 82%), with a high I^2^ statistic (90%).”

Page 12, line 223

“The treatment-seeking proportion of those with lifetime treatment history for prescription OUD was 29% (95% CI: 27%, 31%) (see Appendix H), with no heterogeneity (I^2^=0%).”

Page 12, line 233

“The treatment seeking percentage for those with prescription OUD over the past 12-months was 16% (95% CI: 15%, 17%) (see Appendix I), with no heterogeneity (I^2^= 0%).”

Page 13, line 257

**“**The I^2^ statistic reduced from 97% to 69% with removal of the outliers (14, 18, 19, 32, 35).”

Page 13, line 266

“The pooled percentage of treatment seeking was 40% (95% CI: 22%, 59%), with significant heterogeneity (I^2^= 98%).”

Page 14, line 277

“Hence the pooled proportion is the same as the base case, 21% (95% CI: 16%, 27%, I^2^=98%).”

**11) Unclear why there are numbered commented in the meta-analysis sensitivity results.**

The numbers have been removed. A sentence has been included on page 12, line 241 stating:

“Due to high heterogeneity, three sensitivity analyses were undertaken. These were to remove the outliers, separate adults from adolescents and lastly the metaXL sensitivity analysis.”

**12) Meta-analysis result figures in the supplemental materials are cut off on the right side. Please revise to include complete figure.**

All Meta-analysis result figures, both in the manuscript (Figure 2 and Figure 3) and the supplementary materials, have been redone so the right side is no longer cut off.

**Reviewer #2: I found this to be a very interesting and well written article. My comments are relatively minor:
- Abstract. Please review the Objective sentence. There appears to be a word missing. Maybe "behaviour"?**

Page 2, line 26 - Behaviour was missing. Abstract object line now states:

“To determine treatment seeking behaviour in those with opioid use disorder (OUD) in the United States of America.”

**Reviewer #3: This manuscript sought to understand the “gap” in treatment-seeking behavior among individuals with opioid use disorder in high-income countries. While a noble effort and important topic, there are significant concerns in the presentation of the information, as well concerns about biases in the data collection that warrant further understanding.

The title is a little misleading in that the review doesn’t cover “high-income countries” as a whole. Despite that being the intent, the data are nearly entirely focused on the United States, and so there is a potential for readers or others to generalize these results to other high-income countries where there are significant differences in healthcare compared to the United States. Given that the end result in the data, the authors may consider removing the single UK study and focusing the paper on the United States alone, which would still be of interest. The original methodology and research question could still be included, but with the decision given the search results, that the instead the data were limited to the United States.**

This has been addressed in the reviewer 1 comments above. We have removed the Kimegard et al, 2017 study because the study did not appropriately/consistently measure OUD. The review only has studies from the USA included, however the meta-analysis and systematic review was set up to include all high-income countries. It is a limitation that only studies from the USA were available in the literature to be included and a gap in the literature.

**The authors need to provide better context surrounding the complexity of the gap in treatment-seeking for opioid use disorder. The authors note previous research identifying gaps in treatment-seeking for alcohol use disorder, but opioid use disorder involves more complex substance use patterns that make it more difficult to account for treatment-seeking patterns, particularly when comparing 2008, the year of the first study reported in the analysis, to that of recent years.

Most notably, this span of years in the United States is complicated by two issues:

1. In 2008, opioid use predominantly involved prescription opioids, but over the past decade, heroin use drastically increased in the wake of prescription opioid use reductions resulting from prescription-focused interventions.
2. In addition, the provision of buprenorphine was extremely low early on, but has drastically increased, making treatment with buprenorphine not equally accessible across the study time period.

Even today, many treatment centers/programs in the United States, due to our disparate provision of healthcare, do not provide buprenorphine or methadone. According to the 2018 National Survey of Substance Abuse Treatment Facilities, 39.6% provide no pharmacotherapy treatment. Although the authors appear to use broad terms for opioid treatment, the lack of searching for other specific types of treatment like the inclusion of buprenorphine or methadone may have limited the results.**

I have included a sentence in the literature limitations to address this point

Page 23, line 466

“Lastly, although broad terms relating to OUD treatment and pharmacotherapy were used in the search strategy, the only specific treatment types included were methadone and buprenorphine. This means that studies with other specific treatment types, for example behavioural interventions or naltrexone, may have been missed. However, this review is determining the prevalence of treatment seeking to any treatment rather than individual treatment types and therefore the results may not be heavily impacted.”

**In addition, using only articles in English would also have been a significant factor in limiting the results. Not all high-income countries publish all of their articles in English, and suggesting there is not data from high income countries is different than saying there is not data published in English from high income countries.**

We have included a statement in the study limitations to include the absence of non-English published studies.

Page 23, line 457

“Secondly, the review only included studies published in English and therefore important treatment seeking studies published in other languages have been missed.” **It may be helpful to understand the exclusion criteria in the flow chart at a country level. This could help inform readers as to what the current limitations are, or what types of studies are being conducted in other countries, in order to spur more research on this issue in those countries.**

We have included a brief paragraph under a new heading literature gap about the exclusion criteria and relevant country specific data. We have included this on page 21, line 424

“The exclusion criteria for this review includes several exclusion categories that may show where the literature gaps are when studying treatment seeking behaviour in other countries (USA and non-developed countries are excluded). There was one study from the Netherlands where opioid use was not the main substance studied and rather SUD more generally was studied. Similarly, there were two studies from the United Kingdom where OUD was not the main diagnosis and SUD more generally was studied. This emphasises the need for more specific OUD related treatment seeking behaviour. OUD diagnosis was not measured using a reliable diagnostic tool, for example the ICD-10 or the DSM-V, in studies from United Kingdom, Canada and Australia meaning that a diagnosis is based on self-report or other unvalidated measures (e.g., self-report of illicit drug use in the past month or the presence of injecting marks). “ **The authors note that self-reported studies were excluded from the interview. However, they include data collected from NSDUH, which is self-reported. I believe NSDUH was included because DSM criteria are included in the questions, thus offering a way to “meet” diagnostic criteria, but this is still self-report.**

Yes, the treatment seeking component is self-reported in both NSDUH and NESARC. The data used to complete the DSM criteria is also self-reported by the participant, as is the case with any diagnosis. These NSDUH studies were included because participants met the diagnostic criteria of OUD according to the DSM. Other studies that did not use the DSM or ICD diagnostic criteria were excluded because they asked a single question such as, have you ever used heroin? as a proxy for OUD.

Two statements have been added to discuss the introduction of recall bias:

page 22, line 446

“all but one study (32) assessed treatment seeking through self-reporting, which has the potential to introduce recall bias regarding treatment. Furthermore, although only studies with DSM and ICD OUD diagnoses were included in this review, self-reporting is used to input the data for these diagnostic tools, which may introduce recall bias.”

**“Where studies reported both heroin OUD and prescription OUD, prescription OUD was reported in the main analyses while heroin OUD was included in the subgroup analyses?” What was the rationale for this decision? This seems like it could have time-related bias.**

We have changed this and now where studies reported both heroin OUD and prescription OUD both were reported in the main analyses and subgroup analyses if appropriate. The changes in the manuscript are as follows:

Abstract results section on page 2, line 36

Results: “There were 13 quantitative studies included in the systematic review, with all studies being from the United States of America (USA). IVhet models showed that 40% (95% CI: 23%, 58%) and 21% (95% CI: 16%, 26%) sought treatment in their lifetime and past 12 months respectively. Sub-group analysis found that lifetime treatment seeking for prescription OUD, 29% (95% CI: 27%, 31%), was less than for heroin plus combined OUD, 54% (95% CI: 26%, 82%).”

Page 6, line 119

“Where studies reported both heroin OUD and prescription OUD, both results were reported in the main analyses and subgroup analyses if appropriate.”

Page 9, line 187

“Figure 2 shows that 40% (95% CI: 23%, 58%) of people with OUD sought help for OUD treatment in their lifetime. The I^2^ statistic for heterogeneity is 98%, which suggests a considerable amount of difference between the studies. The five studies included in the lifetime treatment seeking base group were cross-sectional studies from online recruitment samples (34) or nationally representative surveys. (15, 18, 20, 35) Two of the studies determined help-seeking behaviour for prescription OUD, (15, 35) one study reviewed OUD help-seeking behaviour for all opioids (18) and the final study reviewed treatment seeking from both heroin and prescription opioids. (20)”

*Appendix c (the RE model) and Appendix D (the doi plot) have both been updated in the supplementary material and the reporting of the LFK index on page 10, line 196:
“The LFK index, which reports publication bias, reported no asymmetry with LFK = -0.14. (see Appendix D)”

Page 11, line 297

“The type of OUD that was present in the included studies were prescription OUD (12, 14, 15), combined OUD (16-19, 36) and one study reported both prescription OUD and heroin OUD. (33) One of the included studies was undertaken in adolescents only (12) and the remaining eight studies were all done in adults. The LFK index for model 2 reported major asymmetry with LFK = -2.01. (see Appendix F)

*Appendix E (the RE model) and Appendix F (doi plot) have both been updated in the supplemental material

page 12, line 245

**“**One outlier was outside the 95% CI range and was removed from the lifetime treatment base case (see Appendix L). (20) The help-seeking percentage, 39%, (95% CI: 23%, 56%) did not change with removal of the outlier, due to the small weight (2%) the outlier had on the overall pooled proportion. I^2^ statistic was 98%, indicating significant heterogeneity.”

*Appendix L outlier model has been updated in the supplemental material

Page 13, line 253

“Four outlier studies were removed from the 12-month treatment seeking base case (14, 18, 19, 36) and in one study the prescription OUD treatment seeking was an outlier, but not the heroin OUD treatment seeking proportion. (33) (appendix M) Removal of the outliers reduced the treatment seeking percentage from 21% to 20% and narrowed the confidence interval. The I^2^ statistic reduced from 97% to 69% with removal of the outliers. (14, 18, 19, 33, 36)

Page 13, line 259

“The included studies were reviewing treatment seeking in those with prescription OUD (12, 15), combined OUD (16, 17) and heroin OUD. (33)”

Page 13, line 270

“One study examined lifetime OUD treatment seeking in adolescents (34) with treatment seeking proportions for both heroin OUD and prescription OUD. The treatment seeking was 35% (95% CI: 17%, 54%, I^2^= 72%). (Appendix O) The study used a single-site sample.”

*Appendix O - Lifetime OUD treatment seeking IVhet base model adolescents only has been updated in the supplemental material

Page 14, line 275

Eight studies were included in this sensitivity analysis for adults only (see Appendix P). One study (12) was not included in this model when compared with the 12-month treatment seeking base case. Hence the pooled proportion is the same as the base case, 21% (95% CI: 16%, 27%, I^2^=97%). The included studies were identifying treatment seeking in prescription OUD, (14, 15, 33) combined OUD (16-19, 36) and one study reported for both heroin OUD and prescription OUD. (33)

*Appendix P in the supplementary material has been updated

Table 1 metaXL sensitivity analysis (page 15, line 296) updated to include the treatment seeking from prescription opioid and heroin OUD in the Subramaniam study

Table 2 metaXL sensitivity analysis (page 16, line 301) updated to include the treatment seeking from prescription OUD and heroin OUD in the Ober 2018 study.

Page 17, line 317

“Treatment seeking for prescription OUD was the lowest (16% for 12-month treatment and 29% for lifetime treatment) and for heroin plus combined OUD was the highest (54% for lifetime treatment).”

**11 of the 14 studies stem from two surveys NESARC and NSDUH, which suggests the results may be more indicative of these two national survey in the United States rather than “treatment-seeking behavior of high-income countries” and so these results would be limited in the same the way the data from these surveys are limited. The authors may consider limiting their paper to just the United States, where a synthesis of this data would be more meaningful.**

Yes, we agree. Thank you for this comment. While our systematic review was not limited to US studies the results show that only studies conducted in the US met the inclusion criteria. Therefore, we have ensured that all our discussion and interpretation of the results is interpreted in this context. We have changed the review to USA only and removed the one UK study (Kimegard et al 2017) because on closer look the study did not fit the inclusion criteria as OUD was not measured in an appropriate/consistent way. Please see the comments above.

***2^ND^ REVISION***

**Reviewer #1: The revised manuscript entitled “Treatment seeking behaviour among people with opioid use disorder in high income countries: A systematic review and meta-analysis” is significantly improved from the original submission. The authors have been comprehensive in their response to prior reviews. I have no additional major critiques. One suggestion would be to state in the abstract that while the search was intended to capture all high income countries only manuscripts from the US met inclusion criteria.**

Thank you for this comment, we have included a line in the results and conclusion section of the abstract to indicate that only US countries met the inclusion criteria.

Results - Page 2, line 36: “There were 13 quantitative studies included in the systematic review, with all studies being from the United States of America (USA).”

Conclusion - Page 2, line 44: “All included studies in this meta-analysis were from the USA. Therefore, this review found that in the USA one in five people with OUD sought OUD treatment in the previous 12 months and two in five people with OUD sought OUD treatment in their lifetime. These results indicate modest levels of treatment seeking for those with OUD in the USA, the reason for this low treatment seeking requires immediate attention and future work.”

**Reviewer #2: Upon re-reviewing this article I realized that the authors seem to use "treatment seeking" and "access to treatment" interchangeably. For instance, the Objective in the Abstract is to "To determine treatment seeking behaviour in those with opioid use disorder (OUD) in the high-income countries" but the authors present access to treatment. I do not think these 2 terms can be used interchangeably. It would be important for the authors to define/how they conceptualize "treatment seeking" and justify why they then move on to using the term "access". Alternatively, please remove the "access to treatment language".**

We have removed all sections that discuss access to treatment and instead used treatment seeking.

These changes are found:

Abstract page 2, line 44: “Conclusion: All included studies in this meta-analysis were from the USA. Therefore, this review found that in the USA one in five people with OUD sought OUD treatment in the previous 12 months and two in five people with OUD sought OUD treatment in their lifetime.”

Page 4, line 63: “An Australian treatment history study of heroin dependence, found that 88% had previously sought treatment in their lifetime. (21)”

Page 6, line 110: “The data extraction sheet included the following categories to describe and compare the studies: author, year, country, sample description, study design, sample size, opioid type, OUD diagnosis tool, participant mean age and gender, type of treatment sought, treatment time frame and percentage or proportion that sought treatment.”

Page 17, line 314: “It is expected that treatment seeking occurs at a higher proportion within a longer timeframe (12 months versus lifetime) and other studies have also found that increased disease duration and severity increase the likelihood of treatment seeking for OUD.”

Page 19, line 363: “Treatment programs, differing health systems, stigma in differing culture as well as stigma surrounding specific drug used all play a role in treatment seeking behaviour.”

Page 22, line 440: “which may lead to differences in treatment seeking behaviours for those with OUD.”

**Please also specify whether any relevant articles published before 2008 appeared in your search. If so, why were they excluded given that there were no year restrictions for your search.**

We had no restrictions on publication year, however there were no relevant studies included in our review that were published prior to 2008. See Appendix B in the supplemental file for included studies.

**Reviewer #3: The authors were very thoughtful in their responses to the reviewers. However, I still have significant concerns about the methodology that were not alleviated with inclusion of several items as limitations, but rather, call into question the appropriateness of the methodology. Some further detail and considerations may be warranted:

The authors note in their response that “only studies from the USA were available in the literature.” I believe there are still a number of issues that arise from this methodology and the objectiveness of this statement. Despite noting it in the limitations, the fact that only English language publications were used discounts a substantial amount of literature in high-income countries, and so continuing to use the title “High-income countries” is misleading to readers. In addition, there are nuances to other high income countries that may not be accounted for. For instance, were detoxification programs included? These are often referred to as simply ‘substance use detoxification’ programs. Would this be accounted for in the search criteria? Were studies on the prescribing of buprenorphine included? The Canadian Institute for Health publishes treatment-seeking statistics (i.e., proportion of Canadians starting opioid therapy) through assessments of prescriptions due to their ability to follow individuals through national health systems. It’s possible that the studies in the United States dominated the results because without nationalized healthcare, we rely on studies, as opposed to utilization of nationalized health systems for this information, which may see publication elsewhere than a study-based journal article. These are not just limitations, but raise questions about the appropriateness of this methodology. This could be why 11 of the 13 articles includes are from just two national studies in the US, which also raise questions about the generalizability of the information provided.**

Search terms regarding treatment seeking were broad such as treatment seeking, help seeking and heroin n2 therapy, which would include and capture all treatments for opioid use disorder, including detoxification episodes if it were for opioids. We did not include substance use detoxification as one of our search terms as we are focusing specifically on opioid use disorders and not on general substance use disorders. It worth noting that we did include specific search terms such as methadone, buprenorphine and suboxone and therefore buprenorphine prescribing was included. Please note the full search strategy can be found in Appendix A in the supplemental file.

Furthermore, there are no systematic reviews or meta-analysis published in the literature that focus on the treatment seeking proportion for substance use disorders more generally. The WHO publish a review focusing on unmet treatment need for mental health disorders and alcohol dependence but this review did not include any substance use disorders (general or specific).

A line in the discussion has been included on page 22, line 430 has been included referring to this:

“Furthermore, there are no reviews looking at treatment seeking in substance use disorders more generally indicating this is an area for further research.”

We have included a comparison between our meta-analysis treatment seeking results for those with OUD and the treatment seeking for alcohol dependence in a meta-analysis from the WHO. This was found on page 17, line 305, but has been removed since (due to comments from the reviewer after this revision) – see 3^rd^ revision below.

“The treatment gap for OUD is therefore comparable to the treatment gap for alcohol dependence and higher than the treatment gap for major depression in the Americas. The 12-month treatment gap for alcohol dependence and major depression in the Americas was 73% and 57% respectively.”

***This statement was in the second revision, but has been removed as per comments from the reviewer in the next revision around the importance of using treatment seeking (which is what our included studies focused on, rather than assuming this is the same thing as treatment gap, which is what the WHO treatment gap meta-analysis focused on***

Regarding other comments and the inclusion of national statistics such as OST national prescribing in Canada, we have followed systematic review/meta-analysis protocol and included grey literature searching in google using the term ““treatment-seeking behaviour in opioid use disorder” and the first 5 pages (50 articles in total)  **As noted in the previous review, NSDUH and NESARC are both interviews where an individual is asked to self-report behaviors, although the authors have updated the manuscript to note that they are asked specific criteria, here off the DSM. This leads to question of what the authors deemed to be “self-report” vs. “diagnosed” OUD that may have led to studies from other countries being excluded. Many other countries likely do not use the DSM, so there may be variations in how opioid use disorder is clinically diagnosed. In France, OUD is primarily treated by general practitioners who prescribe buprenorphine, and like Canada, this is tracked through the French National Health Insurance database. Did the authors only take articles where a diagnostic process was specifically outlined, or was a physician prescribing buprenorphine for OUD enough, on the assumption that the physician would not prescribe buprenorphine if there was not a clinical diagnosis? More information on needs to be provided on what constituted a “clinical diagnosis.”**

Thank you for this comment. We have gone through the studies that were excluded from the meta-analysis due to “no appropriate/consistent measure of OUD ie) self-report, injecting marks.”

There were 8 studies that were excluded because there was “no appropriate/consistent measure of OUD ie) self-report, injecting marks” and these were the following studies (the way in which OUD was “diagnosed” is also given):

1) Larance 2019 – Participants in this study were included if they used a S8 opioid that was given to them legally from a pharmacy in the last 6 weeks.
2) Liebling 2016 – Participants in this study were included if they reported non-medical use of prescriptions opioids in the past 30 days
3) Lundgren 2005 – Participants were included in this study if they reported injecting drugs at least once (primarily heroin) in the year prior
4) Oppenheimer 1988 – Participants were obtained from a drug dependence clinic in South East London. No reference to other inclusion criteria regarding review by doctor, whether they were current or past attendees of the clinic (some participants were interviewed whilst in prison so they could not be current clinic users)
5) Robles 2006 – Participants were included in the study if they reported injecting drugs or smoking crack cocaine during the past 30 days.
6) Stein 2018 – 1 Medicare claim for methadone or buprenorphine, this is inclusion based on treatment rather than a diagnosis and therefore all had experienced treatment and therefore there is no relevant treatment seeking proportion. In this case this study should be changed to the group “no appropriate treatment seeking proportion given.”
7) Stumbo 2017 - minimum of two opioid dependence diagnoses recorded in the EMR during an encounter in the 12 months preceding recruitment. It is common to require two instances of a diagnosis when identifying a sample from the EMR as it greatly reduces misidentification based on a single instance of the diagnosis. As there are electronic medical records present for these participants, this shows that the participants are already likely to be engaged in treatment and therefore this study should also be moved into the “no appropriate treatment seeking proportion given.”
8) Yang 2011 – Participants were included in this study if they reported illicit drug use in the past month.

Studies 1 to 3, study 5 and study 8 all report opioid use in a period of time and there is no assessment by a doctor to indicate the participants have OUD. These studies should stay in the exclusion criteria “no appropriate/consistent measure of OUD i.e.) self-report, injecting marks.”

Study 4 includes participants if they were seen at a dependence clinic, however there is no reference to participants having seen or diagnosed with OUD by a doctor. Furthermore, the participants could be current or past users of the clinic indicating previous but not current use of opioids. This study should stay in the exclusion criteria “no appropriate/consistent measure of OUD i.e.) self-report, injecting marks.”

Study 6 and 7 involve Medicare treatment claims or opioid diagnoses on electronic medical records. This indicates that all participants have already had engagement with the healthcare system and therefore these studies should be removed from the category “no appropriate/consistent measure of OUD i.e.) self-report, injecting marks” and included in “no appropriate help-seeking numerical value related to treatment seeking.” Therefore, we have changed the PRISMA and removed 2 papers from the “no appropriate/consistent measure of OUD i.e.) self-report, injecting marks” and this category now has 7 papers in it and the “no appropriate help-seeking numerical value related to treatment seeking” category now has 150 papers. Prisma has been updated, see figure 1.

We have included an additional author in the paper, who helped with revision comments and review. She is Professor Maree Teesson, director of the Matilda Centre for Research in Mental Health and Substance Use, Sydney University. I have included her in the unblinded title page.

***3^rd^ REVISION***

**The editor stated that: “According to the reviewers, the revised version of the manuscript seems to be inappropriate for publication. Particularly, most of the methodological issues, which have been raised by the Reviewer 3, were not resolved.”**

This was the letter that we sent to the editor to ask for an appeal to the paper being rejected:

While we normally respect editorial decisions regarding paper rejection, we must say we feel that this decision for the current manuscript really does require further explanation. We feel the paper adheres to the principles of publication for Plos One, which are largely centred around methodological rigour. The paper has been reviewed by three separate reviewers and has been revised twice by us. Upon the first revision we were told that reviewer #1 and reviewer #2 generally agree with the publication of the paper, but that reviewer #3 raised issues surrounding search criteria terms, national prescribing statistics in Canada and how OUD was deemed “diagnosed” versus “self-reported”, that needed to be further addressed. The second revision therefore focused on reviewer #3 comments, which we have addressed, and we believe we have resolved. However, in this final rejection letter reviewer 3 did not give any comments. We strongly believe that we have written a methodologically and technically sound systematic review and meta-analysis, which follows the PRISMA guidelines that is the requirement for publication in PLOS ONE and have addressed the reviewer 3 comments in our second revision. It is helpful if you can provide us with further details as to why our manuscript was rejected as we feel we have indeed addressed areas of major technical concern with the manuscript.

**In the rejection letter received from you, we have seen reviewer #2 state: “I appreciate the authors taking the time to address my concerns. After this round of revision I must say these concerns have only been partially addressed. Namely, there continues to be confusion when discussing treatment seeking and unmet treatment needs. For instance, in the Conclusion of the Abstract the authors state that: "This review found that one in five people with OUD sought OUD treatment in the previous 12 months and two in five people with OUD sought OUD treatment in their lifetime. These results indicate a significant unmet treatment need for those with OUD, the reason for this low treatment seeking requires immediate attention and future work." To me, there is a logical gap about drawing conclusions on treatment gaps when the results presented pertain to treatment seeking. This argument would make sense if data presented pertained to people seeking treatment but not receiving it. We could then talk about an unmet need. I think it might be wise to revise the conclusion and focus on how we can move individuals toward treatment seeking. This misalignment is present throughout the manuscript.”**

Thank you for this comment, we totally agree with reviewer #2 that treatment seeking cannot be used to determine treatment gaps. We have now double-checked carefully throughout the manuscript to ensure that treatment seeking has been used in the manuscript.

This has been addressed in the following areas in our manuscript:

Abstract, Conclusion, Page 2, line 44:

“All included studies in this meta-analysis were from the USA and indicate modest levels of treatment seeking for those with OUD. In particular, this review found that in the USA one in five people with OUD sought OUD treatment in the previous 12 months and two in five people with OUD sought OUD treatment in their lifetime. Further research is urgently required to explore the barriers and facilitators that can improve this low treatment seeking in those with OUD.”

Presently, there are no systematic reviews or meta-analysis that focus on treatment seeking behaviour in those with OUD, substance use disorders or mood disorders. There is a published meta-analysis by the WHO that determines the treatment gaps in alcohol dependence and other mental health. This was used in the previous revision of our paper to compare treatment gaps in the WHO review paper with our review on treatment seeking. However, as reviewer #2 has stated this is not appropriate and therefore this comparison has been removed. Instead we have included in the discussion a comparison between our meta-analysis treatment seeking results for those with OUD and the treatment seeking for mood disorders and substance use disorders from a single study (not a systematic review or meta-analysis) published by the WHO in the discussion. The comparison now compares like with like, ie) treatment seeking to treatment seeking. This is found on page 17, line 304:

“Our systematic review and meta-analysis found that one in five people (20%) or one in two people (50%) with OUD sought help during the previous 12-months and during their lifetime in the US, respectively. It is noteworthy that it is the first review including a meta-analysis to quantify the proportion of people with OUD seeking treatment. A study conducted by the World Health Organization (WHO) found that the 12-month treatment seeking proportion for mood disorders and substance use disorders in the USA was 35% and 10% respectively. The treatment seeking proportion for OUD in our study is therefore slightly less than the treatment seeking for mood disorders and slightly higher than the treatment seeking for substance use disorders. The lower treatment seeking levels in those with substance use disorders could be due to lack of established effective and available treatments for other substance use disorders such as cocaine and methamphetamine compared to opioid replacement treatments for OUDs. ”

Page 24, line 475

“These results indicate low levels of treatment seeking for those with OUD in the USA. The reasons for these modest levels of treatment-seeking behaviour and barriers to treatment in this population require immediate attention and future work should be undertaken in this area.”

**Furthermore, as per the staff editor comment “We would strongly recommend that you pay special attention to the reviewers’ comments regarding the inclusion of US-based studies only, and we would suggest to carefully discuss this issue and the associated limitations in the abstract, results and conclusions sections. Please bear in mind that our publication criteria state “The data presented in the manuscript must support the conclusions drawn. Submissions will be rejected if the interpretation of results is unjustified or inappropriate, so authors should avoid overstating their conclusions”.**

The following changes have been made:

Abstract page 2, line 36:

“Results: There were 13 quantitative studies included in the systematic review, with all studies being from the United States of America (USA).”

Abstract page 2, line 44:

“Conclusion: All included studies in this meta-analysis were from the USA. Therefore, this review found that in the USA one in five people with OUD sought OUD treatment in the previous 12 months and two in five people with OUD sought OUD treatment in their lifetime. These results indicate modest levels of treatment seeking for those with OUD in the USA, the reason for this low treatment seeking requires immediate attention and future work.”

Results section, Page 8, line 168:

“All 12 studies were conducted in the USA.”

Discussion section, page 17, line 303:

“Our systematic review and meta-analysis found that one in five people (20%) or one in two people (50%) with OUD sought help during the previous 12-months and during their lifetime in the USA, respectively.”

Discussion section, page 18, line 348:

“All studies in this systematic review were from the USA. It is noteworthy that other high-income countries report proportion of seeking treatment in substance use disorders in general, but not specific to opioid use disorders.”

Discussion section, page 19, line 354:

“Therefore this review, **with studies only from the USA**, found that less people with an OUD sought treatment (one in five people with OUD sought treatment in the past 12 months) than those with SUD in Australia (one in three people with SUD sought treatment in the past 12 months).

Literature limitations section, page 22, line 439:

“All included studies were from the USA, which means the results cannot be generalised to other countries due to the notable differences in health care systems, which may lead to differences in treatment seeking behaviours for those with OUD. In the USA healthcare is a market driven business opportunity whereas in other countries, for example the UK, healthcare is a public good. (101) It would be ideal to include other countries with varying health systems in the review, however presently these studies are not available in the literature.”

Study limitations section, page 23, line 455:

“Although this review included studies that were conducted in developed countries, all studies were from the USA. Therefore, results are not transferable to countries other than the USA. More research is required on the treatment seeking levels in other countries. Secondly, the review only included studies published in English and therefore important treatment seeking studies published in other languages have been missed.”

Conclusion section, page 24, line 475:

“These results indicate low levels of treatment seeking for those with OUD in the USA. The reasons for these modest levels of treatment-seeking behaviour and barriers to treatment in this USA population require immediate attention and future work should be undertaken in this area. Treatment-seeking behaviour in the USA was found to be lowest in adolescents and adults with prescription OUD and highest in adults with heroin OUD.”
